# A preliminary study on predicting the prognosis of testicular torsion based on indocyanine green-guided near-infrared fluorescence imaging

**DOI:** 10.3389/fped.2026.1770405

**Published:** 2026-02-06

**Authors:** Xiaomeng Liu, Yi Xu, Yuqian Ren, Qing Sun, Xiaomeng Cui, Cheng Huang, Dongsheng Bai

**Affiliations:** Department of Urology, Capital Center for Children’s Health, Capital Medical University, Beijing, China

**Keywords:** fluorescence imaging, indocyanine green, testicular atrophy, testicular torsion, testicular viability

## Abstract

**Background:**

Testicular viability is the primary basis for selecting the surgical approach during orchiopexy for testicular torsion (TT); however, there is currently a lack of methods that can objectively assess testicular viability intraoperatively. The purpose of this study is to evaluate testicular viability and predict postoperative testicular outcomes using indocyanine green-guided near-infrared fluorescence (ICG-NIRF) imaging during surgery for TT.

**Methods:**

We retrospectively reviewed pediatric patients treated for TT at our hospital between January 2024 and December 2024. Intraoperative ICG-NIRF imaging was used to assess testicular viability. Cases were classified into three types based on the testicular fluorescence patterns: Type A (extensive parenchymal fluorescence), Type B (fluorescence limited to the tunica vasculosa), and Type C (no fluorescence). Follow-up ultrasounds were performed at 1, 3, and 6 months postoperatively to record testicular volume and blood flow, and to calculate the testicular atrophy index (TAI).

**Results:**

This study enrolled a total of 19 pediatric patients. The cases were distributed as follows: 8 cases of Type A, 8 cases of Type B, and 3 cases of Type C. At the 6-month postoperative follow-up, Type A testes all showed normal blood flow signals on ultrasound, seven cases exhibited no atrophy, and one case had mild atrophy, the TAI [median (*P_25_*, *P_75_*)] was 4.75% (−0.72, 9.34). Among the 8 Type B testes, ultrasound showed normal blood flow signals in 1 case, reduced blood flow in 5 cases, and absent blood flow in 2 cases. All cases experienced severe atrophy, with a TAI [median (*P_25_*, *P_75_*)] of 86.95% (83.83, 97.31). Among the 3 Type C testes, ultrasound revealed reduced blood flow signals in 1 case and absent blood flow signals in 2 cases. All cases demonstrated severe atrophy, with a TAI [median (*P_25_*, *P_75_*)] of 88.15% (86.65, 90.56). There was a statistically significant difference in the TAI on comparison of Type B and Type C with Type A, respectively (*p* = 0.003 and *p* = 0.030).

**Conclusion:**

The application of ICG-NIRF imaging during surgical intervention for TT can effectively assess testicular viability and predict the prognosis of TT.

## 1 Introduction

Testicular torsion (TT) is one of the common acute scrotal conditions in urology. It results from abnormal anatomical structures of the testis and spermatic cord or from increased testicular mobility, leading to twisting of the spermatic cord, which subsequently results in impaired testicular blood supply (1, 2). The prognosis of TT is closely related to the duration and degree of torsion; therefore, prompt diagnosis and early treatment are essential for preserving testicular function (3). Reported studies indicate that the orchiectomy rate during TT surgery ranges from 20% to 60%, while the long-term postoperative testicular atrophy rate ranges from 30% to 67% (3). Currently, intraoperative assessment of testicular viability primarily relies on observing testicular color after detorsion and evaluating parenchymal bleeding following incision (4, 5). However, neither method can accurately and objectively reflect testicular blood perfusion. Consequently, the choice of surgical approach remains largely dependent on the surgeon's subjective judgment.

Indocyanine green-guided near-infrared fluorescence (ICG-NIRF) imaging has been used in surgery to assess tissue and organ blood perfusion (6–8). The principle involves using near-infrared light at a specific wavelength to excite indocyanine green (ICG), causing it to emit fluorescence, which is then captured by a specialized device and converted into a real-time visual display (9). Our previous study demonstrated the feasibility of using ICG-NIRF imaging in assessing testicular viability during TT surgery (10).

This study is designed to explore the clinical value of ICG-NIRF imaging for assessing testicular viability and predicting TT prognosis by applying this technique to visualize intraoperative testicular blood perfusion.

## 2 Materials and methods

### 2.1 Study design and research subjects

**Figure 1:**
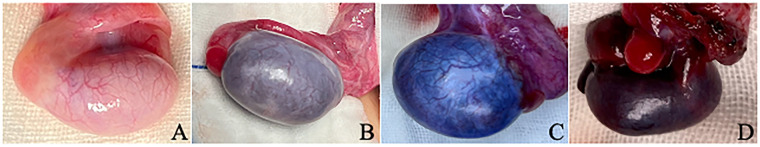
Testicular color grading. **(A)** Grade 1 (Pink) **(B)** Grade 2 (Light Blue) **(C)** Grade 3 (Dark Blue) **(D)** Grade 4 (Black).

**Figure 2:**
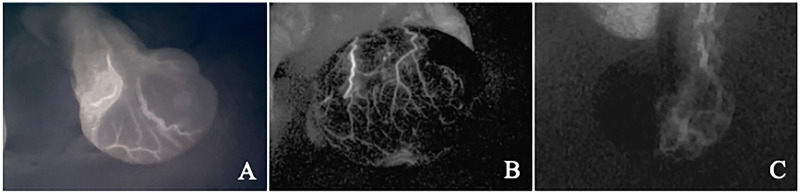
Testicular fluorescence imaging patterns. Type A: extensive parenchymal fluorescence **(A)** Type B: fluorescence limited to the tunica vasculosa without parenchymal fluorescence **(B)** Type C: absence of fluorescence in both the tunica albuginea and parenchyma **(C)**.

This retrospective study reviewed pediatric patients with TT who underwent surgical treatment in the department of urology at Capital Center for Children's Health, Capital Medical University, between January 2024 and December 2024. The inclusion criteria were as follows: (a) age 1–18 years; (b) intraoperative use of ICG-NIRF imaging to assess testicular viability, with subsequent orchiopexy on the affected side; and (c) no history of prior testicular surgery. The exclusion criterion was incomplete clinical data. Collected clinical data included age, torsion duration, affected side, degree of torsion, testicular color after detorsion, testicular fluorescence imaging results, complications, postoperative testicular volume, and postoperative testicular blood flow. Based on testicular color, the torsioned testes were classified into four grades: Grade 1 (pink), Grade 2 (light blue), Grade 3 (dark blue), Grade 4 (black) (Figure 1). Based on testicular fluorescence imaging, the torsioned testes were categorized into three types: Type A (widespread parenchymal fluorescence), Type B (fluorescence limited to the tunica vasculosa without parenchymal fluorescence) and Type C (absence of fluorescence in both the tunica albuginea and parenchyma) (Figure 2).

### 2.2 Surgical method and postoperative follow-up

Following the induction of general anesthesia, the patient was placed in the supine position. A transverse incision was made in the mid-scrotum on the affected side to expose the testis, and the direction and degree of torsion were recorded. The torsioned testis was manually detorsioned and wrapped with warm saline-soaked gauze for 10 min, after which its color was recorded. Subsequently, a transverse incision was made in the midline of the contralateral hemiscrotum to expose the healthy testis. ICG was administered intravenously (0.2 mg/kg), and the testicular fluorescence imaging findings were recorded.

Based on the findings from our previous study (10), if fluorescence was observed in the affected testis within 5 min (Type A or B), orchiopexy was performed. Otherwise (Type C), the tunica albuginea was incised for further evaluation based on the presence of parenchymal bleeding. If active bleeding from the testicular parenchyma was observed, orchiopexy was performed; if not, orchiectomy was performed. Regardless of the outcome for the affected testis, prophylactic orchiopexy of the contralateral testis was performed in all cases.

Postoperatively, all patients underwent ultrasonographic follow-up at 1, 3, and 6 months to assess testicular volume and blood flow. Severe and mild testicular atrophy were defined as a Testicular Atrophy Index (TAI) of ≥50% and 20%–50%, respectively. Testicular volume was calculated using the formula: Length×Width×Thickness×π/6. TAI was calculated as: (volume of contralateral testis - volume of affected testis)/volume of contralateral testis×100%.

### 2.3 Statistical analysis

Continuous data were presented as median (range) or median (*P_25_*, *P_75_*), and comparisons were performed using the Kruskal–Wallis test, with *post-hoc* Dunn's test for multiple comparisons. Categorical data were expressed as *n* (%). All statistical analyses were conducted using SPSS software (version 27.0), and all figures were generated using GraphPad Prism software (version 10.0). A *p*-value < 0.05 was considered statistically significant. In figures, significance levels are denoted as follows: * for *p* < 0.05 and ** for *p* < 0.01.

## 3 Results

A total of 19 pediatric patients (median age: 11 years, range: 1–14) were enrolled. The median torsion duration was 13 h (range: 3–48 h) and the median torsion degree was 360° (range: 180°–1,080°). The affected side was left in 14 cases and right in 5 cases. Based on intraoperative testicular fluorescence imaging, cases were classified as Type A (*n* = 8), Type B (*n* = 8), and Type C (*n* = 3). Among the Type C cases, two showed bleeding upon incision of the testicular parenchyma, for the remaining case without observed bleeding, the parents strongly requested testicular preservation, and orchiopexy was performed accordingly. According to the testicular color grading after detorsion, there were 5 cases of Grade 1, 5 cases of Grade 2, 8 cases of Grade 3, and 1 case of Grade 4. Among the Grade 3 cases, two exhibited bleeding upon incision of the testicular parenchyma. No bleeding was observed upon incision of the testicular parenchyma in one case classified as Grade 4 (Table 1).

**Table 1:** Clinical data.

Classification	Age [years, median (range)]	Torsion duration [hours, median (range)]	Torsion degree [Degree, median (range)]	Affected side (*n*, %)		Parenchymal bleeding of the testis	
				Left	Right	Bleeding	No bleeding
Testicular fluorescence imaging patterns							
Type A (*n* = 8)	11 (1, 13)	15 (4, 48)	450 (180, 1,080)	4 (50.0)	4 (50.0)	–	–
Type B (*n* = 8)	12 (4, 14)	14 (3, 27)	360 (180, 720)	7 (87.5)	1 (12.5)	–	–
Type C (*n* = 3)	12 (12, 14)	6 (3, 41)	360 (360, 540)	3 (100.0)	0	2	1
Testicular color grading							
Grade 1 (*n* = 5)	11 (3, 13)	20 (4, 48)	360 (180, 1,080)	2 (40.0)	3 (60.0)	–	–
Grade 2 (*n* = 5)	9 (1, 13)	14 (3, 48)	270 (180, 540)	3 (60.0)	2 (40.0)	–	–
Grade 3 (*n* = 8)	12 (4, 14)	13 (3, 41)	360 (180, 720)	8 (100.0)	0	2	–
Grade 4 (*n* = 1)	12	3	360	1 (100.0)	0	–	1

**Figure 3:**
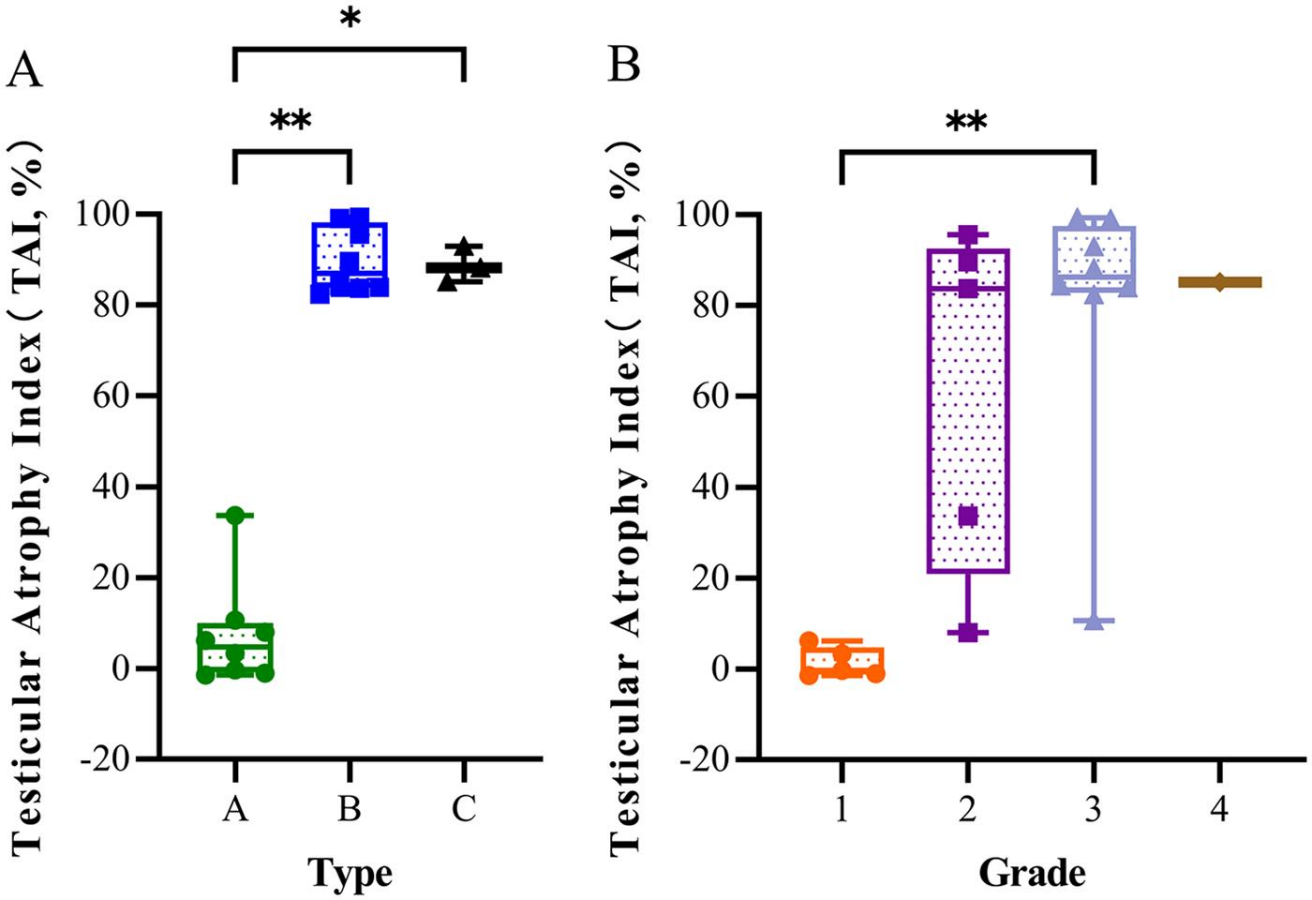
Testicular atrophy at 6 months postoperatively. **(A)** Relationship between testicular fluorescence imaging patterns and testicular atrophy index. **(B)** Relationship between testicular color grading and testicular atrophy index. *P < 0.05, **P ≤ 0.01.

At the 6-month postoperative follow-up, outcomes were analyzed according to testicular fluorescence imaging classification. Among the 8 Type A testes, ultrasound revealed normal blood flow signals in all cases. Seven testes showed no atrophy and one had mild atrophy with a TAI [median (*P_25_*, *P_75_*)] of 4.75% (−0.72, 9.34) [The TAI for all testes except Grade 4 are presented as median (*P_25_*, *P_75_*)]. Among Type B testes, ultrasound revealed normal blood flow signals in 1 testis, reduced blood flow signals in 5 testes, and absent blood flow signals in 2 testes. All testes exhibited severe atrophy, with a TAI of 86.95% (83.83, 97.31). Among the 3 Type C testes, ultrasound showed reduced blood flow signals in 1 and absent blood flow signals in 2 testes. All testes developed severe atrophy, with a TAI of 88.15% (86.65, 90.56). Statistical comparisons of TAI showed significant differences between Type B and Type A (*p* = 0.003) and between Type C and Type A (*p* = 0.030) (Figure 3A). According to testicular color grading, all 5 Grade 1 testes showed normal blood flow on ultrasound and no atrophy, The TAI was −0.38% (−1.05, 3.29). Among the 5 Grade 2 testes, ultrasound showed normal blood flow signals in 3 testes and reduced blood flow signals in 2 testes, 1 testis showed no atrophy, 1 testis had mild atrophy, and 3 testes exhibited severe atrophy, with a TAI of 83.72% (33.72, 89.55). Among the 8 Grade 3 testes, normal blood flow signals were observed by ultrasound in 1 testis, reduced blood flow signals were observed in 4 testes, no blood flow signals were observed in 3 testes, 1 testis showed no atrophy, and 7 testes developed severe atrophy. The TAI was 86.25% (83.15, 96.00). Grade 4 testis demonstrated absence of blood flow on ultrasound and severe atrophy, with a TAI of 85.15%. Meanwhile, the comparison of TAI between Grade 3 and Grade 1 showed a statistically significant difference (*p* = 0.008) (Figure 3B; Table 2).

**Table 2:** Outcomes of the affected testis at 6 months postoperatively.

Classification	Testicular Blood Flow (*n*, %)			Testicular Atrophy (*n*, %)			TAI [%, median (*P*_25_, *P*_75_)]
	Normal	Reduced	No	No	Mild	Severe	
Testicular fluorescence imaging patterns							
Type A (*n* = 8)	8 (100.0)	0	0	7 (87.5)	1 (12.5)	0	4.75 (−0.72,9.34)
Type B (*n* = 8)	1 (12.5)	5 (62.5)	2 (25.0)	0	0	8 (100.0)	86.95 (83.83,97.31)
Type C (*n* = 3)	0	1 (33.3)	2 (66.7)	0	0	3 (100.0)	88.15 (86.65,90.56)
Testicular color grading							
Grade 1 (*n* = 5)	5 (100.0)	0	0	5 (100.0)	0	0	−0.38 (−1.05,3.29)
Grade 2 (*n* = 5)	3 (60.0)	2 (40.0)	0	1 (20.0)	1 (20.0)	3 (60.0)	83.72 (33.72,89.55)
Grade 3 (*n* = 8)	1 (12.5)	4 (50.0)	3 (37.5)	1 (12.5)	0	7 (87.5)	86.25 (83.15,96.00)
Grade 4 (*n* = 1)	0	0	1 (100.0)	0	0	1 (100.0)	85.15

## 4 Discussion

Rapid and accurate assessment of testicular viability during TT surgery presents a significant clinical challenge. Due to the inaccuracy and lack of standardization in existing evaluation methods, viable testes may be incorrectly removed (11). Most surgeons tend to preserve the torsioned testis whenever possible to avoid erroneous removal of still-viable tissue. However, this approach also leads to the preservation of numerous necrotic testes, thereby increasing the risks of postoperative testicular pain, infection, and secondary surgeries. Therefore, compared with preserving viable testes, removing necrotic testes or those confirmed to develop severe postoperative atrophy is equally important.

The color of the testis after detorsion is frequently used for intraoperative viability assessment, though a unified testicular color grading standard remains lacking. In our study, all Grade 1 testes showed no postoperative atrophy, while all Grade 4 testes developed severe atrophy. Among Grade 2 and 3 testes, outcomes varied with cases showing no atrophy, mild atrophy, and severe atrophy. These results demonstrate the difficulty in predicting testicular outcomes based solely on color for Grade 2 and 3 testes. We attribute this primarily to the subjective nature of surgeons' color assessment and the absence of unified objective criteria. Additionally, variations in ambient lighting conditions may cause color perception discrepancies, further compromising surgical judgment. Consequently, we consider testicular viability assessment based solely on color to be unreliable, particularly for Grade 2 and 3 testes in our study.

Assessment of testicular viability by incising the testis to observe parenchymal bleeding was first proposed by Arda et al. (5). Although this method reduces the surgeon's subjective influence compared to testicular color observation, several limitations remain. Firstly, the observed bleeding may originate from venous or tunica vasculosa (5), potentially misleading the surgeon's viability assessment. For instance, in this study, two testes showed bleeding upon incision but ultimately developed severe atrophy. Secondly, the requirement for testicular incision may cause additional damage to viable testes, as observed in Type A and Grade 1 testes in our study. Furthermore, some scholars suggest combining testicular incision with tunica vaginalis flap coverage to alleviate compartment pressure (12), though its efficacy in restoring testicular viability remains uncertain (13).

The application of ICG-NIRF imaging technique offers potential improvements to this situation. This method enables objective visualization of testicular blood perfusion during TT surgery (10, 14, 15). ICG produces rapid fluorescence (16), with normal testes demonstrating fluorescence within one minute after intravenous injection (10). After injection, ICG binds to plasma proteins and is subsequently cleared by the liver and excreted via the bile (17). Recent expert consensus recognizes both fluorescence imaging technique and ICG application as highly safe (18, 19). However, it must be emphasized that ICG contains sodium iodide and is contraindicated in individuals with iodine allergy (19).

In our study, with the exception of one case of mild atrophy, all Type A testes showed no postoperative atrophy, while all Type B and C testes developed severe atrophy. These outcomes align closely with the intraoperative ICG-NIRF imaging findings. ICG-NIRF imaging provides an intuitive visualization of blood perfusion in the torsioned testis. We believe that the presence or absence of fluorescence development in the testicular parenchyma after intraoperative ICG injection can serve as an objective indicator for assessing testicular viability. Extensive parenchymal fluorescence (Type A) indicates robust perfusion and warrants preservation. For Type B testes, although fluorescent images are visible in the tunica vasculosa, this alone is not sufficient to confirm parenchymal viability. As Arda et al. (5) stated, bleeding observed from the tunica albuginea after incision cannot be used as an indicator of parenchymal viability. However, some scholars suggest that Type B testes may have a favorable prognosis (15); therefore, surgical management for these testes should be carefully considered. Consistent with our previous findings (10), the absence of fluorescence in both the tunica albuginea and testicular parenchyma (Type C) suggests severe hemorrhage and necrosis in the testicular tissue. This observation aligns with the finding that all Type C testes in the present study eventually developed severe atrophy.

It is noteworthy that although ICG-NIRF imaging holds significant potential for assessing testicular viability and predicting the prognosis of TT, only a preliminary evaluation of its clinical value is currently available. The following limitations of this study should be considered. First, the limited sample size may introduce random errors into the results, particularly for Type C testes. Although all Type B and Type C testes in this study eventually developed severe atrophy, it must be emphasized that this research represents only an initial exploration of ICG-NIRF imaging for evaluating testicular viability and predicting TT prognosis. For Type B and Type C testes, combining other assessment methods is recommended to comprehensively evaluate the viability of the torsioned testis and avoid unnecessary removal of still-viable tissue. Additionally, for Type C testes, cases requiring orchiectomy were excluded to allow postoperative ultrasound follow-up of the affected side, which may have introduced selection bias. Second, this study did not account for the potential effects of torsion duration and degree on testicular prognosis, which may have led to an overestimation of the association between ICG-NIRF imaging patterns and outcomes. Finally, the follow-up period was relatively short, which may have underestimated the risk of atrophy in Type A testes. Further follow-up of these pediatric patients will be conducted to validate the technique's effectiveness. We believe that with the inclusion of large-scale, multicenter, and prospective studies in the future, ICG-NIRF imaging technique will gain greater clinical value in assessing testicular viability and predicting TT prognosis, ultimately evolving into a standardized method.

## 5 Conclusion

Taken together, intraoperative ICG-NIRF imaging for TT objectively visualizes testicular blood perfusion, thereby guiding surgical decision-making. It aids in preserving viable testes while avoiding the retention of non-viable tissue. Furthermore, it can predict postoperative outcomes based on the testicular fluorescence patterns. This technique holds promise as a standardized method for the intraoperative assessment of testicular viability and the prediction of postoperative testicular outcomes in TT.

## Data Availability

The original contributions presented in the study are included in the article/Supplementary Material, further inquiries can be directed to the corresponding author.
